# Right Upper Lobe Torsion after Right Lower Lobectomy: A Rare and Potentially Life-Threatening Complication

**DOI:** 10.1155/2018/2146458

**Published:** 2018-12-23

**Authors:** Takeo Nakada, Yo Tsukamoto, Mitsuo Yabe, Takeyuki Misawa, Tadashi Akiba, Takashi Ohtsuka

**Affiliations:** ^1^Department of Surgery, The Jikei University Kashiwa Hospital, 163-1 Kashiwashita, Kashiwa, Chiba 277-8567, Japan; ^2^Department of Surgery, The Jikei University School of Medicine, Nishishinbashi 3-19-18, Minatoku, Tokyo 105-8471, Japan

## Abstract

An 84-year-old woman was referred to our institution with suspected right lung cancer. Subsequently, she underwent thoracoscopic right lower lobectomy without mediastinal lymph node dissection. Postoperatively, she complained of dyspnea and developed arterial oxygen desaturation after 12 h and acute respiratory failure (ARF). An emergency chest computed tomography revealed the right upper bronchial stenosis with hilar peribronchovascular soft tissue edema because the middle lung lobe had been pushed upward and forward and the right upper lung lobe had twisted dorsally. Emergency bronchoscopy revealed severe right upper bronchial stenosis with an eccentric rotation and severe edema. The bronchia stenosis was successfully treated with glucocorticoids and noninvasive positive pressure ventilation for ARF.

## 1. Introduction

 Lung torsion after lobectomy is a potentially life-threatening complication due to the possible development of acute respiratory failure (ARF). Here we report the case of a woman who underwent thoracoscopic right lower lobectomy without lymph node dissection for advanced f-stage IIB lung cancer and subsequently developed right upper lobe torsion causing ARF 12 h postoperatively. The upper lobe twisting just after right lower lobectomy is a rare potentially life-threatening complication.

## 2. Case Report

**Figure 1:**
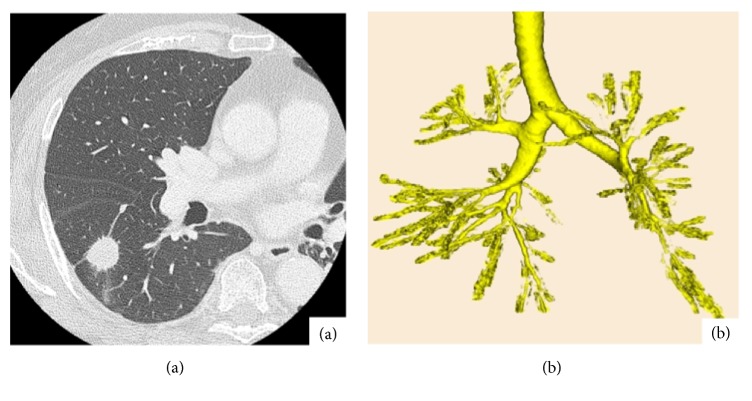
(a) Chest computed tomography (CT) showing a 1.8 cm, irregular nodular lesion in the right lower pulmonary lobe. (b) Three-dimensional CT showing normal bronchial anatomy.

**Figure 2:**
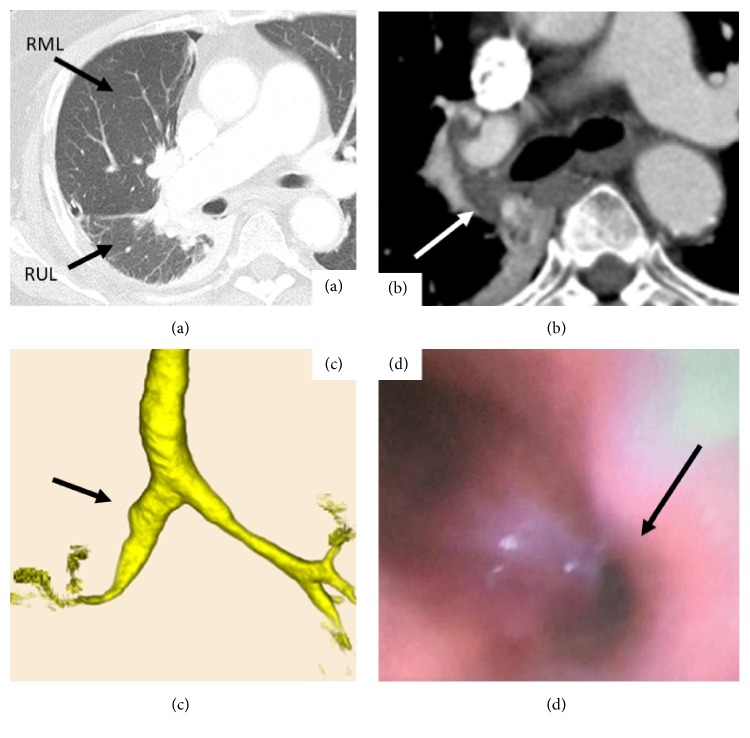
(a) An emergency chest computed tomography (CT) showed the middle lung lobe had been pushed upward and forward, and the right upper lung lobe had twisted dorsally. (b) The right upper bronchus with hilar peribronchovascular soft tissue edema (arrow). (c) Three-dimensional CT scan of bronchial stenosis (arrow). (d) Emergency bronchoscopy showing severe right upper bronchial stenosis (arrow). RUL: right lower lobe; RML: right middle lobe.

**Figure 3:**
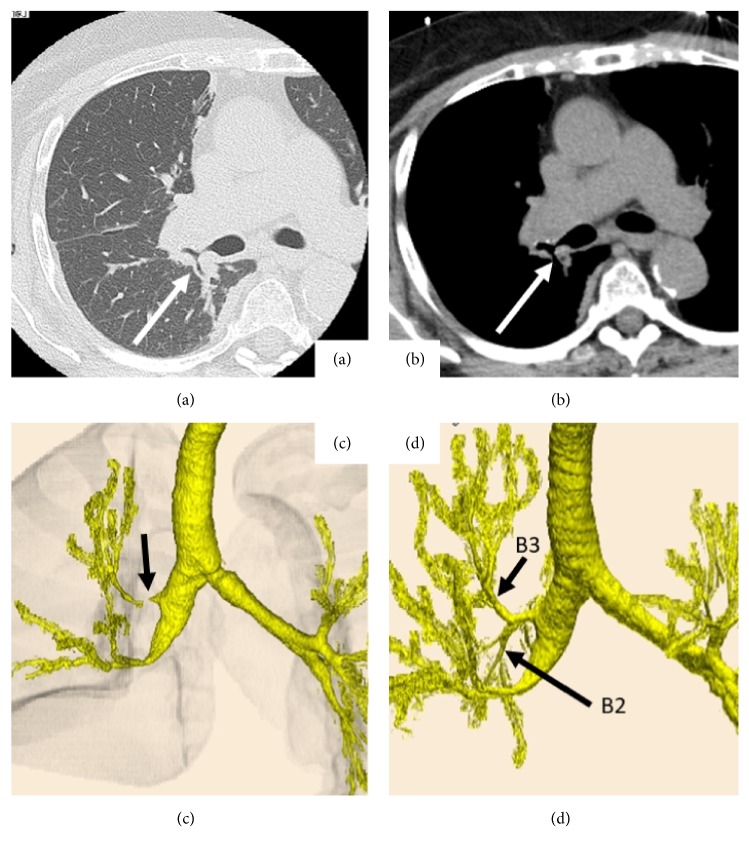
Follow-up chest computed tomography (CT) on postoperative day (POD) 3 with the (a) lung window, (b) mediastinal window, and (c) three-dimensional image revealing improvement of the hilar peribronchovascular soft tissue edema and right upper bronchial stenosis (arrows). (d) 3D-CT on POD 14 showing the counterclockwise rotation of right upper lung lobe and improvement of the right upper bronchial stenosis.

An 84-year-old woman with angina, diabetes mellitus (DM), hypertension, and Alzheimer's disease was referred to our institution for suspected right lung cancer. Physical examination revealed the following: body height, 150 cm; weight, 68.4 kg; and body mass index, 30.4. Chest computed tomography (CT) revealed a 1.8 cm nodular lesion with an ill-defined margin in the right lower lobe, suggesting lung cancer without metastasis (Figure 1(a)). Three-dimensional CT revealed normal bronchial anatomy (Figure 1(b)). Her preoperative vital capacity was 1.77 L as assessed using a spirogram, and the forced expiratory volume in 1 s was 1.35 L. Subsequently, we performed thoracoscopic right lower lobectomy without mediastinal lymph node dissection. The anesthetic and operative times were 189 and 92 min, respectively, with minimal blood loss. The total amount of intraoperative fluid replacement was 1000 mL. Final pathological finding was adenocarcinoma with hilar lymph node metastasis diagnosed as pT1bN1M0 (p-stage IIB according to the 8^th^ IASLC classification criteria) [1]. Extubation was safely performed in the operating room, and she was followed up in the intensive care unit. However, postoperatively, she complained of dyspnea without chest pain and developed arterial oxygen desaturation 12 h postoperatively. Oxygen saturation reduced to 86% despite the administration of 10 L/min oxygen, corresponding to a PaO_2_ of 54 mmHg. An emergency chest computed tomography (CT) revealed the right upper bronchial stenosis with hilar peribronchovascular soft tissue edema (PSTE) because the middle lung lobe had been pushed upward and forward, and the right upper lung lobe had twisted dorsally (Figures 2(a) and 2(b)). Three-dimensional CT scan showed severe bronchial stenosis (Figure 2(c)). Emergency bronchoscopy revealed severe right upper bronchial stenosis with an eccentric rotation and severe edema (Figure 2(d)). Echocardiography and electrocardiography revealed a cardiac ejection fraction of 55% and normal diameter of the inferior vena cava, thus ruling out ischemic heart disease. Subsequent emergency blood tests revealed normal hepatorenal function and serum albumin level. She was diagnosed with localized right upper bronchial obstruction with bronchial edema and hilar PSTE due to right upper lobe torsion after right lower lobectomy. There was no evidence of venous congestion, hemorrhagic infarction, necrotic findings, increased pleural effusion, or atelectasis. Therefore, we decided conservative treatment as primary care. ARF was treated using noninvasive positive pressure ventilation for 2 days and 40 mg methylprednisolone injection for 3 days. A follow-up chest CT on postoperative day (POD) 3 revealed improvement of the right upper bronchial stenosis; she subsequently received 30 mg oral predonine for 7 days (Figures 3(a)–3(c)). 3D-CT on POD 14 showed the counterclockwise rotation of right upper lung lobe but obvious improvement of stenosis of the bronchus (Figure 3(d)). The chest tube was removed on POD 1. She was discharged on POD 16, after recovery. At the 4-month follow-up, she exhibited good health without any evidence of right upper bronchial stenosis.

## 3. Discussion

Lung torsion (LT) is a very rare but potentially life-threatening complication and can be caused by pulmonary resection, tumor, or trauma [2–4]. Hennink had reported that the incidence of LT after pulmonary resection is less than 0.4% [5]. In reference to lobar torsion after lung resection, middle lobar torsion after right upper lobectomy (41.0%) and left lower lobar torsion after left upper lobectomy (23.1%) were mainly reported. Then, the right upper lobe (RUL) torsion after lung resection after right anterior segmentectomy (2.6%) and right middle lobectomy (7.7%) had been reported. The LT-related mortality rate was 8.3% [6]. In this literature, we describe the first case of RUL torsion causing ARF after right lower lobectomy.

Depending on the symptoms, such as congestion or infarction, incomplete LT also needs surgical intervention. Particularly, in incomplete cases, it is important to rule out other possible causes of bronchial edema. Although bronchial edema is a rare surgical complication of lobectomy, the potential risk of progression to ARF should be recognized. Bronchial edema may be caused by excessive transfusion, cardiac dysfunction, barotrauma to the bronchus due to positive pressure ventilation, or circulating vasoactive mediators due to surgical reactive or ischemic changes. Although cardiac ultrasonography performed by a cardiovascularspecialist should have been employed to rule out acute cardiac failure, no evidence of such cardiac failure causing edema could be identified as a hepatorenal disorder or hypoalbuminemia. Both CT and bronchoscopy are the modalities of choice for prospective diagnosis of LT. Clinicians should search inversion of the vascular pattern, congestion, or infarction of the affected lung by enhanced chest CT [6, 7]. Bronchoscopy was an alternative examination, and the most suggestive bronchoscopic findings included bronchial occlusion and fish mouth orifice [5, 8]. In this case, we suspected bronchial edema due to RUL incomplete torsion by these multiple examinations to rule out other pathophysiology.

Moreover, PSTE develops secondary to conditions such as pulmonary lymphangitic carcinomatosis, lymphoproliferative disorders, hydrostatic pulmonary edema, pneumonia, interstitial pulmonary emphysema, and interstitial hemorrhage [9–11]. In this case, the exacerbation factors of bronchial edema with PSTE, except for the reactive change of LT, were suspected to be as follows: (1) ipsilateral lymphatic vessels were damaged due to lobectomy and impaired lymphatic drainage; (2) local capillary permeability at the affected peribronchovascular soft tissue may have increased as a result of the surgical invasion; and (3) DM could affect the pathogenesis of endothelial damage in diabetic micro- and macroangiopathy around the twisted right upper bronchus [12]. Our case had no evidence of venous congestion, hemorrhagic infarction, necrotic findings, increased pleural effusion, or atelectasis. Finally, glucocorticoids and noninvasive positive pressure ventilation successfully treated the patient. Prompt and effective treatment should be provided to prevent critical pulmonary failure under the strict monitoring.

## 4. Conclusion

We report a case of right upper lobe torsion causing ARF after right lower lobectomy. This is a rare and potentially life-threatening surgical complication following lobectomy. The patient was successfully treated using glucocorticoids and noninvasive positive pressure ventilation for ARF. Finally, glucocorticoids and noninvasive positive pressure ventilation successfully treated the patient. Prompt and effective treatment should be provided to prevent critical pulmonary failure under the strict monitoring.
